# Eight Americas: Differences in Asian Communities Are Important

**DOI:** 10.1371/journal.pmed.0040041

**Published:** 2007-01-30

**Authors:** Linda Silka, Robin Toof, Dorcas Grigg-Saito

The article “Eight Americas: Investigating Mortality Disparities across Races, Counties, and Race-Counties in the United States” [1] reports on what the authors describe as racial differences in mortality. The authors analyze what they label the “eight Americas” (i.e., Asian; Northland low-income rural white; Middle America; low-income whites in Appalachia and the Mississippi Valley; Western Native American; Black Middle America; Southern low-income rural black; high-risk urban black). In contrast to other races, “Asian” is treated as a single homogenous category. Income and geographical differences are not considered. The authors point out that they have likely collapsed across differences with the “Asian” category, but they go on to report their results as if such differences are inconsequential. They are not. With regard to health disparities, such differences are particularly important.

Consider recent findings that speak to health differences within the nominal category of “Asian.” As a part of the Center for Disease Control and Prevention's Racial and Ethnic Approaches to Community Health (REACH) 2010 program [2], a survey was conducted in 2001–2002 with a sample of Vietnamese in several counties in California and Cambodians in Lowell, Massachusetts (the second largest Cambodian community in the US). Comparing the results to the national 2002 Behavior Risk Factor Surveillance System survey that aggregates all Asian responses, the Koch-Weser et al. data indicate that the educational level and income of Cambodians and Vietnamese were substantially lower than all Asians, and that Cambodians and Vietnamese were three times more likely than other Asians to not have visited a doctor in the past year due to financial reasons. In addition, in comparison to all Asians or the general population, higher proportions of Cambodian and Vietnamese men reported smoking (50.4% and 30.4% respectively compared to 14.7% of aggregated Asians), and Cambodian and Vietnamese of both genders reported eating fewer vegetables (16.4% and 11.1%). And in the case of important chronic health problems such as diabetes, only 47.7% of Cambodians surveyed reported having their cholesterol checked and 41.9% reported having a hemoglobin A1c test conducted if they had diabetes.

A 2002 representative survey of Cambodian adults over age 25 in Lowell, Massachusetts [3] found that Cambodians were more likely to report poor health than other Massachusetts residents (9% compared to 2%). Cambodian women and elders were much more likely to have experienced days of poor physical health (6.5 days on average for women and 8 days those over 50). A quarter of the Cambodian elders were symptomatic for depression, with the rate rising to 43% among women 50 and over. Although only 6% reported being uninsured, 23% wanted to see a doctor in the last year but could not, and 44% did not because of transportation problems.

In short, existing findings indicate how diverse the health data can be within the overall category of “Asian.” The authors are to be applauded for their recognition of how misleading it can be to treat the categories of “blackness” or “whiteness” in undifferentiated ways. Unfortunately they have failed to extend that same understanding to the analysis they select for the category of Asians. As researchers and policy makers use the “Eight Americas” study to guide their efforts, the result could well be misleading interpretations that do a disservice to those very groups within the “Asian” category who face daily struggles with significant health problems and poor access to health care.
